# Miniature Schnauzers under primary veterinary care in the UK in 2013: demography, mortality and disorders

**DOI:** 10.1186/s40575-019-0069-0

**Published:** 2019-02-15

**Authors:** Dan G. O’Neill, Charlotte Butcher, David B. Church, Dave C. Brodbelt, Alex G. Gough

**Affiliations:** 1 Pathobiology and Population Science, The Royal Veterinary College, Hawkshead Lane, North Mymms, Hatfield, Herts AL9 7TA UK; 2 Clinical Sciences and Services, The Royal Veterinary College, Hawkshead Lane, North Mymms, Hatfield, Herts AL9 7TA UK; 3 Bath Veterinary Referrals, Rosemary Lodge, Wellsway, Bath, BA2 5RL UK

**Keywords:** Prevalence, Canine, VetCompass, Primary-care, Epidemiology, Breed predisposition, Pedigree

## Abstract

**Background:**

Individual dog breeds are often reported as predisposed to specific breed-related disorders but reliable epidemiological data on disease prevalence are sparse. The Miniature Schnauzer in the UK is a popular small breed dog that is often considered as relatively healthy and long-lived, but is this really true? This study aimed to use data from the VetCompass™ Programme at the Royal Veterinary College to characterise the demography, mortality and common disorders of the general population of Miniature Schnauzers under veterinary care in the UK.

**Results:**

The study population of 455,557 dogs from 304 clinics in the VetCompass™ database under veterinary care during 2013 included 3857 Miniature Schnauzers (0.85%). For dogs with data available, 1771 (56.9%) were neutered and 1893 (49.2%) were females. Mean adult bodyweight overall was 9.9 kg (SD 2.2 kg) and median longevity was 11.6 years (IQR 9.3–13.1, range 0.5–17.0). The most prevalent fine-level precision disorders recorded were periodontal disease (*n* = 343, prevalence 17.4, 95% CI: 15.7–19.1), obesity/overweight (164, 8.3, 95% CI: 7.1–9.6), anal sac impaction (114, 5.8, 95% CI: 4.8–6.9), vomiting (100, 5.1, 95% CI% 4.1–6.1) and otitis externa (99, 5.0, 95% CI% 4.1–6.1). The most prevalent grouped-level precision disorders were dental (*n* = 378, prevalence: 19.2, 95% CI: 17.5–21.0), enteropathy (270, 13.7, 95% CI: 12.2–15.3), cutaneous (250, 12.7, 95% CI: 11.2–14.2) and aural (197, 10.0, 95% CI: 8.7–11.4).

**Conclusions:**

This study provides generalisable evidence on the demography, longevity and most prevalent disorders in the Miniature Schnauzer breed in the UK. Awareness of common diseases and breed predispositions can support evidence-based policies to improve breed health, guide veterinary surgeons when producing differential diagnosis lists, and assist owners when purchasing or caring for their pets.

## Plain English summary

Concerns are regularly raised about the health of purebred dogs and their reportedly high level of inherited disease predisposition. However, despite perceptions that we know much about the health of individual breeds, there is actually quite little reliable information on the frequency of specific common diseases in individual breeds. The Miniature Schnauzer is a popular small breed dog which is often considered as relatively healthy and long-lived. But is this really true? This study aimed to describe the frequency of diagnosis of the most common diseases affecting Miniature Schnauzers by exploring large numbers of anonymised clinical records from first opinion veterinary practices in the UK that participate in the VetCompass™ disease surveillance programme. Miniature Schnauzers comprised 3847 out of 455,557 dogs in the study (0.85%). The average body weight was 9.9 kg and the average lifespan was nearly 12 years. The most common conditions recorded were dental disease (17.4%), obesity/overweight (8.3%), anal sac blockage (5.8%), vomiting (5.1%) and ear infections (5.0%). These results can help breed organisations and breeders to improve decision-making to enhance breed health, can guide veterinarians on which diseases are more common in the breed when making diagnoses, can help prospective owners when choosing a suitable pet for their needs, and can help Miniature Schnauzer owners to better care for their own pets.

## Background

The Schnauzer dog breed has been recorded in Germany since the fifteenth century, with the recognisable form depicted in artwork by Albrecht Durer in 1492 [1]. The Standard Schnauzer was originally used in Germany as a drover’s dog, pulling and guarding carts, and also as a rat-catcher and a herder of sheep, cattle and hogs [1]. The breed was miniaturised in the nineteenth century by reportedly outcrossing with the Affenpinscher [1, 2] to create a house pet that retained the looks and temperament of the standard sized breed [1]. The UK Kennel Club breed standard describes the Miniature Schnauzer as a “sturdily built, robust [and] sinewy” dog, “nearly square, (length of body equal to height at shoulders)” [3]. The Miniature Schnauzer is classified within the Utility Group by the UK Kennel Club, but in the Terrier Group of the American Kennel Club [3, 4].

The UK Kennel Club registered 5611 Miniature Schnauzers from 243,290 new registrations overall (2.3% of all new registrations) in 2017 [5]. The popularity level of the pedigree subset of the Miniature Schnauzer has been very consistent in recent years, with between 5152 and 5924 Miniature Schnauzers registered in the UK annually from 2007 to 2017 (comprising 2.0 to 2.5% of all registrations) [5]. Although the Kennel Club only registers around 30% of the overall UK dog population (i.e. the pedigree subset of the breed) [6], these figures suggest that the popularity of the Miniature Schnauzers has remained relatively stable over the last decade. In the US, the Miniature Schnauzer is currently the 17th most popular breed registered by the American Kennel Club [7]. However, there is little information available on the popularity of the Miniature Schnauzer in the wider UK dog population although such information is important to truly understand the wider welfare issues facing the breed [8].

It has been reported for over half a century that individual dog breeds are predisposed to specific breed-related disorders [9] and the health issues of purebred dogs are increasingly recognised as a major welfare issue [8, 10]. The Kennel Club’s Breed Watch scheme ‘serves as an early warning system to identify points of concern for individual breeds’ [11]. The Miniature Schnauzer is classified as Breed Watch Category 1 meaning that “No visible health concerns have been reported by judges or breed club(s)/council” [12] and currently has no points of concern relating to health “identified for special attention by judges, other than those covered routinely by the Kennel Club Breed Standard.” [11]. Although undoubtedly useful, the Breed Watch system primarily aims to assist show judges in identification of visible abnormalities and therefore does not cover conditions which are unrelated to conformation but may still adversely affect health nor does it collect data on the subset of the wider population of Miniature Schnauzers that does not fall under the provenance of the Kennel Club. Therefore, information on the health of the general population for this breed is important to understand issues relating to the overall UK population of Miniature Schnauzers.

In relation to the epidemiology of dog health, a clear and important distinction needs to be made between prevalence and predisposition. Prevalence is an absolute value that defines the overall frequency of a condition whereas predisposition is a relative value that describes the prevalence in one subgroup in comparison to some other subgroup e.g. comparing disease levels between breeds or sexes or in comparison to the overall population of dogs [13]. It is perfectly possible for a disease to have a high prevalence and be very relevant to the health of a breed but yet that breed need not show a breed predisposition (i.e. this high prevalence may be no higher than the overall average for all dogs). Conversely, it is perfectly possible for a breed to show disease levels with a high predisposition relative to all dogs but yet that predisposed disease need not be a high breed priority (e.g. if the prevalence or severity of the disease is still extremely low even at high levels of predisposition) [14].

Breed predisposition suggests some form of heritable aetiological component for that disease but this often defies explanation by simple mendelian genetics based on a binary predisposed/non-predisposed conceptual model [14]. Alternatively, it may be useful to view the probability of many disease predispositions as a continuum influenced by a complex web of causation including genetic, epigenetic, environmental and even social effects such as the purpose for which the owner keeps the dog/breed [15, 16]. Breed predispositions can vary geographically between, and even within, countries across which closed breeding populations of the same breed may show widely differing disorder frequencies [17]. Breed predisposition can also vary temporally, possibly increasing in frequency as a particular conformational characteristic which is linked to the disease becomes more fashionable [18] or decreasing in frequency as breeding schemes aimed at genetic disease control become more effective [19]. A comprehensive survey of breed predispositions across 200 dog breeds identified 44 reported disease predispositions worldwide over several decades in the Miniature Schnauzer [14] including primary hyperlipidaemia [20], primary hypothyroidism [21], diabetes mellitus [22] and portosystemic shunt [23]. However, reliable data on disease frequency within a specific geography and period are needed to optimise decision-making on disease prioritisation for that specific local and temporal population [14, 24–27].

Data extracted directly from practice management systems (PMS) of veterinary general practices have potential to reveal reliable and useful perspectives of the real-world epidemiology of disease [8, 28]. Primary-care clinical data benefit by including all animals and all diagnosed cases under veterinary care, while clinical records merged from hundreds of practices benefit from high statistical power and reduced selection bias [26]. This study aimed to use data from the VetCompass™ Programme at the Royal Veterinary College [29] to characterise the demography, mortality and common disorders of the general population of Miniature Schnauzers under veterinary care in the UK during 2013. The study placed special focus on exploring sex-related differences in demography and health. These results could provide a reliable framework to assist primary-care clinicians with preventive and diagnostic prioritisation as well as assist breed clubs and regulators to reform breeding practices that can ultimately contribute to improved health and welfare of Miniature Schnauzers.

## Materials and methods

The study population included all dogs under primary veterinary care at clinics participating in the VetCompass™ Programme during 2013. Dogs under veterinary care were defined as those with either a) at least one electronic patient record (EPR) (free-text clinical note, treatment or bodyweight) recorded during 2013 or b) at least one EPR recorded before 2013 and at least one EPR after 2013. Animals that received active veterinary care before and after 2013 were assumed to remain under the care of that practice during 2013 even if no active clinical care was sought during this period. The VetCompass™ Programme collates de-identified EPR data from primary-care veterinary practices in the UK for epidemiological research [29]. Data fields available to VetCompass™ researchers for each dog included a unique animal identifier along with species, breed, date of birth, sex, neuter status and bodyweight, and clinical information from free-form text clinical notes and treatment with relevant dates.

A cross-sectional study design derived from cohort clinical data of dogs registered at participating practices was used to estimate the one-year period prevalence of the most commonly diagnosed disorders [30]. Sample size calculations estimated that 1900 dogs would need to be sampled from a population of 3857 dogs to report a disorder with 2.5% expected prevalence to a 0.5% margin of error [31]. Ethics approval was obtained from the RVC Ethics and Welfare Committee (reference number 2016/U403).

Dogs recorded specifically as Miniature Schnauzer breed at their most recent entry in the veterinary practice management system were categorised as Miniature Schnauzer and all remaining dogs were categorised as non-Miniature Schnauzer. Breed terms are generally assigned within veterinary practice management systems based on consensus between the owners and the veterinary clinical teams and the assigned terms can be updated over time with the aim of improving validity. *All-age Bodyweight* (Kg) described all available bodyweight and date combinations and were used to generate lifetime growth curves; individual animals could contribute differing counts of bodyweight events. *Adult Bodyweight* (Kg) described the mean bodyweight recorded from all bodyweight data for dogs aged over 18 months and was categorised into 5 groups (< 8.0 kg, 8.0 to < 10.0 kg, 10.0 to < 12.0 kg, 12.0 to < 14.0 kg, ≥ 14.0 kg). N*euter* described the status of the dog (entire or neutered) at the final EPR. *Age* described the age (years) at the final date under veterinary care during 2013 (December 31st, 2013) and was categorised into 7 groups (< 2.0 years, 2.0 to < 4.0 years, 4.0 to < 6.0 years, 6.0 to < 8.0 years, 8.0 to < 10.0 years, 10.0 to < 12.0 years, ≥ 12.0 years).

The clinical records of a simple random sample of 1972/3857 (51.1%) Miniature Schnauzers were reviewed manually in detail to extract the most definitive diagnoses recorded for each disorders that existed during 2013 [32]. Elective (e.g. neutering) or prophylactic (e.g. vaccination) clinical events were not included. No distinction was made between disorders that were pre-existing to 2013 compared with those that were incident (i.e. newly diagnosed) during 2013. Disorders described within the clinical notes using presenting sign terms (e.g. ‘vomiting’ or ‘vomiting and diarrhoea’), but without a formal clinical diagnostic term being recorded, were included using the first sign listed (e.g. vomiting). Mortality data (recorded cause, date and mechanism of death) were extracted on all deaths at any date during the available EPR data.

The extracted diagnosis terms were mapped to a dual hierarchy of diagnostic precision for analysis: fine-level precision and grouped-level precision as previously described [32]. Briefly, fine-level precision terms described the original extracted terms at the maximal diagnostic precision recorded within the clinical notes (e.g. *inflammatory bowel disease* would remain as *inflammatory bowel disease*). Grouped-level precision terms mapped the original diagnosis terms to a general level of diagnostic precision (e.g. *inflammatory bowel disease* would map to *gastro-intestinal*).

**Fig. 1:**
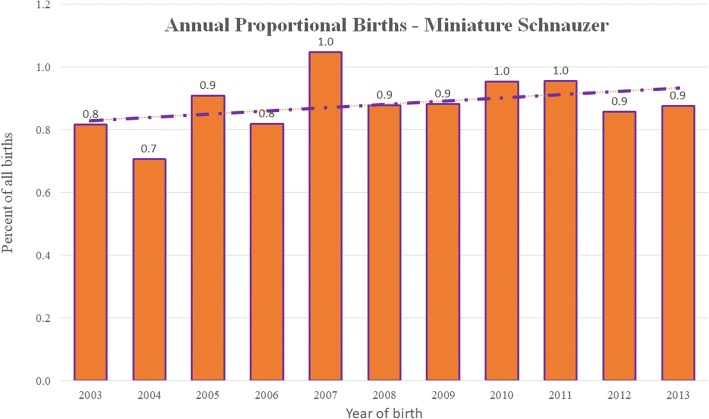
Annual proportional birth rates (2003–2013) for Miniature Schnauzers (*n* = 3857) among all dogs (*n* = 455,557) attending UK primary-care veterinary clinics participating in the VetCompass™ Programme

**Fig. 2:**
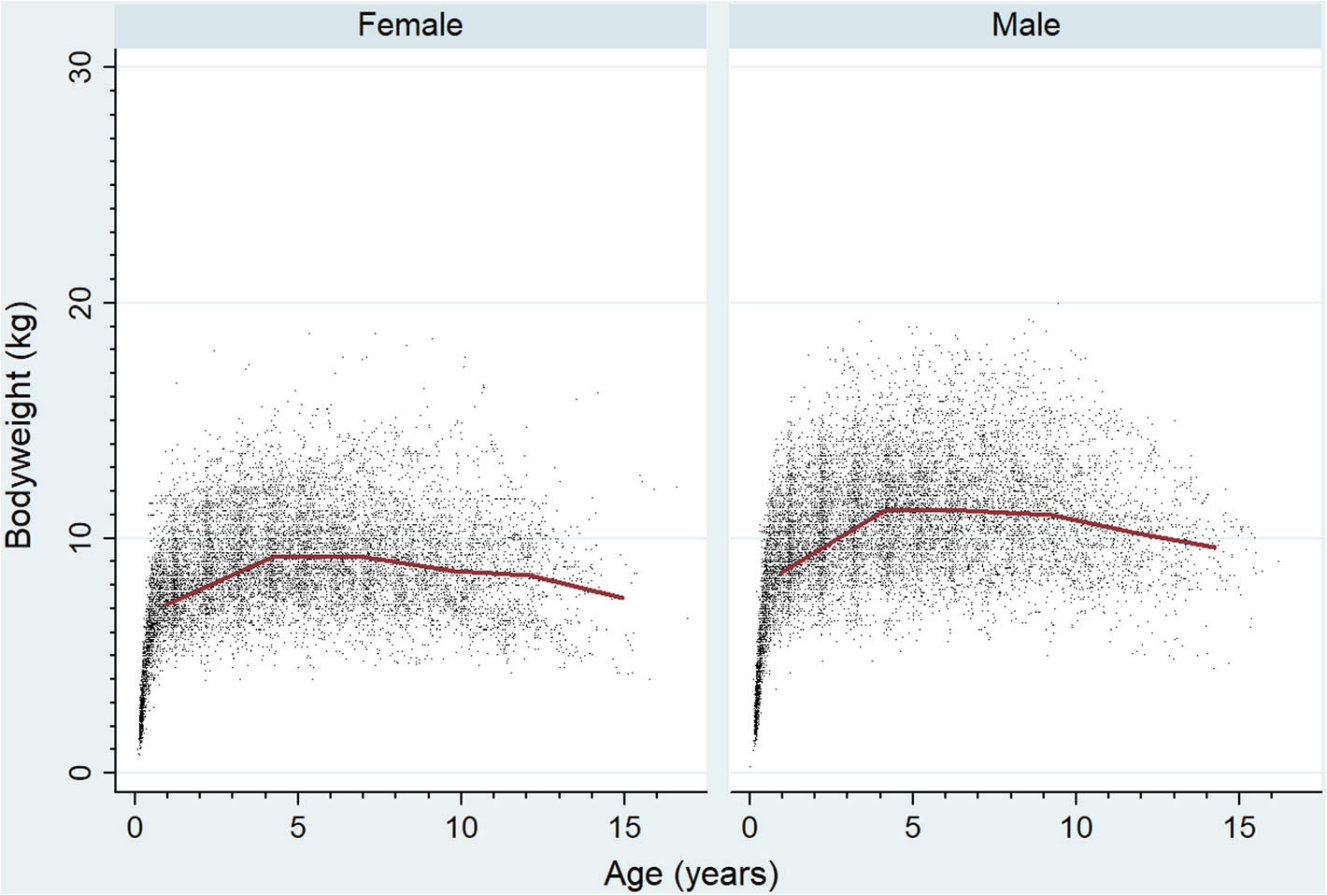
Bodyweight growth curves overlaid with a cross medians line plot for female and male Miniature Schnauzers attending UK primary-care veterinary clinics participating in the VetCompass™ Programme. (12,443 bodyweight values from 1284 females and 12,545 bodyweight values from 1327 male)

Following data checking and cleaning in Excel (Microsoft Office Excel 2013, Microsoft Corp.), analyses were conducted using Stata Version 13 (Stata Corporation). The sex, neuter status, age and adult bodyweight for Miniature Schnauzers under veterinary care during 2013 were described. Annual proportional birth rates described the relative proportion of Miniature Schnauzers compared with all dogs that were born in each year from 2003 to 2013 from the cohort that were under veterinary care in 2013 (Fig. 1). All-age bodyweight data with their associated dates were used to generate individual bodyweight growth curves for male and female Miniature Schnauzers by plotting age-specific bodyweights and were overlaid with a cross medians line plot using the Stata *mband* command (Fig. 2).

One-year (2013) period prevalence values were reported along with 95% confidence intervals (CI) that described the proportion of dogs with a diagnosis during 2013. The numerator described the count of dogs affected with the disorder and the denominator described the count of dogs in the sample tested (1972). The CI estimates were derived from standard errors based on approximation to the normal distribution for disorders with ten or more events [33] or the Wilson approximation method for disorders with fewer than ten events [34]. Prevalence values were reported overall and also separately for males and females. The chi-square test was used to compare categorical variables (e.g. sex) and the Students t-test or Mann-Whitney U test to compare continuous variables (e.g. longevity) as appropriate [33]. Statistical significance was set at the 5% level.

## Results

### Demography and mortality

The study population of 455,557 dogs from 304 clinics in the VetCompass™ database under veterinary care during 2013 included 3857 (0.85%) Miniature Schnauzers. Of the 3857 Miniature Schnauzers with information available for the relevant variable, there were 1771/3111 (56.9%) neutered animals and 1893/3846 (49.2%) females. Females were more likely to be neutered than males (*n* = 964/1553, 62.1% versus *n* = 807/1548, 52.1%, *P* < 0.001). Mean adult bodyweight overall was 9.9 kg (standard deviation [SD] 2.2 kg). The mean adult bodyweight of males (10.8 kg, SD 2.2 kg) was heavier than for females (9.0 kg, SD 1.8 kg) (*P* < 0.001). The median age at December 31, 2013 of the Miniature Schnauzers overall was 3.8 years (interquartile range [IQR] 1.7–6.9, range 0.1–17.2) (Table 1). Data completeness varied across the variables assessed: sex 99.7%, age 99.0%, neuter 80.7% and all-age bodyweight 67.7%. Annual proportional birth rates showed minor changes over time in the popularity of Miniature Schnauzers in the UK, ranging from 0.7% of all puppy births in 2004 to 1.0% in 2011 (Fig. 1). Annual birth rates were based on dogs that were still alive during 2013 and make an assumption of similar longevities between the Miniature Schnauzers and the remaining population. Given the median longevity value for Miniature Schnauzers of 11.7 years reported in the next section and the median longevity of 12.0 years previously reported for dogs overall, this assumption appears to be safe [35]. The median bodyweight across all ages for males (10.1 kg, IQR: 8.4–11.9, range: 0.3–20.0) was higher than for females (8.4 kg, IQR: 7.0–9.9, range: 0.8–18.7) (*P* < 0.001). Bodyweight growth curves based on 12,443 bodyweight values from 1284 females and 12,545 bodyweight values from 1327 males showed that Miniature Schnauzer puppies grow rapidly during their first year with a trend to further weight gain up to four years of age (Fig. 2).

**Table 1:** Demography of Miniature Schnauzers under primary veterinary care at practices participating in the VetCompass™ Programme in the UK from January 1st, 2013 to December 31st, 2013 (*n* = 3857)

Variable	Category	Count^a^	Percent
Sex	Female	1893	49.2
	Male	1953	50.8
Female neuter	Entire	589	37.9
	Neutered	964	62.1
Male neuter	Entire	741	47.9
	Neutered	807	52.1
Female adult bodyweight (aged ≥18 months) (kg)	< 8.0	338	28.8
	8.0 to < 10.0	531	45.2
	10.0 to < 12.0	236	20.1
	12.0 to < 14.0	55	4.7
	≥ 14.0	14	1.2
Male adult bodyweight (aged ≥18 months) (kg)	< 8.0	99	8.4
	8.0 to < 10.0	358	30.4
	10.0 to < 12.0	407	34.6
	12.0 to < 14.0	220	18.7
	≥ 14.0	93	7.9
Age (years)	< 2.0	1085	28.4
	2.0 to < 4.0	890	23.3
	4.0 to < 6.0	623	16.3
	6.0 to < 8.0	504	13.2
	8.0 to < 10.0	335	8.8
	10.0 to < 12.0	227	5.9
	≥ 12.0	156	4.1

^a^Results from dogs with available data

There were 86 deaths recorded during the study. The median age at death of Miniature Schnauzers overall was 11.6 years (IQR 9.3–13.1, range 0.5–17.0). The median age at death of females (11.6 years, IQR 9.3–13.5, range 0.5–17.0, *n* = 42) did not differ to males (11.6 years, IQR 9.2–12.9, range 3.3–15.4, *n* = 44) (*P* = 0.465). Of the 75/86 (87.2%) dogs with a recorded cause of death, the most common causes of death described at a grouped-precision level were neoplasia (*n* = 11, 14.7%), collapse (10, 13.3%), mass-associated disorder (8, 10.7%) and brain disorder (8, 10.7%) (Table 2).

**Table 2:** Grouped causes of mortality in Miniature Schnauzers with a recorded cause of death under primary-care veterinary at UK practices participating in the VetCompass™ Programme from January 1st, 2013 to December 31st, 2013 (*n* = 75)

Grouped disorder term	Count	Percent	95% CI
Neoplasia	11	14.7	7.6–24.7
Collapse	10	13.3	6.6–23.2
Mass-associated disorder	8	10.7	4.7–19.9
Brain disorder	8	10.7	4.7–19.10
Renal disease	6	8.0	3.0–16.6
Enteropathy	5	6.7	2.2–14.9
Anorexia	3	4.0	0.8–11.2
Endocrine disorder	3	4.0	0.8–11.3
Other	21	28.0	18.2–39.6
Total	75		

### Disorder prevalence

The EPRs of a random selection of 1972 (51.1%) from the overall 3857 Miniature Schnauzers were manually examined to extract all recorded disorder data for 2013. There were 1345/1972 (68.2%) Miniature Schnauzers with at least one disorder recorded during 2013 while the remaining 627 (31,8%) had no disorder recorded and either presented for prophylactic management only or did not present at all during 2013. The median annual disorder count per Miniature Schnauzer during 2013 was 1 disorder (IQR 0–2, range 0–9) and did not differ between the sexes (*P* = 0.734).

The study included 2784 unique disorder events recorded during 2013 that encompassed 281 distinct fine-level disorder terms. The most prevalent fine-level precision disorders recorded were periodontal disease (*n* = 343/1972, prevalence 17.4, 95% CI: 15.7–19.1), obesity/overweight (164/1972, 8.3, 95% CI: 7.1–9.6), anal sac impaction (114/1972, 5.8, 95% CI: 4.8–6.9), vomiting (100/1972, 5.1, 95% CI% 4.1–6.1) and otitis externa (99/1972, 5.0, 95% CI% 4.1–6.1). Females had a higher proportion of animals with a diagnosis than males for 3 of the 25 most common fine-level precision disorders (periodontal disease, obesity/overweight and heart murmur) while males had higher proportion of animals with a diagnosis than females for two fine-level precision disorders (diarrhoea and claw injury), although some of these differences were numerically small (Table 3).

**Table 3:** Prevalence of the most common disorders at a *fine-level of diagnostic precision* recorded in Miniature Schnauzers (*n* = 1972) attending UK primary-care veterinary practices participating in the VetCompass™ Programme from January 1st, 2013 to December 31st, 2013

Fine-level disorder	Count	Overall prevalence %	95% CI*	Female prevalence %	Male prevalence %	*P*-value
Periodontal disease	343	17.4	15.7–19.1	19.6	15.4	**0.014**
Obesity/overweight	164	8.3	7.1–9.6	10.2	6.4	**0.002**
Anal sac impaction	114	5.8	4.8–6.9	5.2	6.4	0.251
Vomiting	100	5.1	4.1–6.1	4.8	5.4	0.545
Otitis externa	99	5.0	4.1–6.1	5.2	4.9	0.749
Ear disorder	97	4.9	4.0–6.0	4.4	5.5	0.256
Heart murmur	82	4.2	3.3–5.1	5.5	2.9	**0.003**
Diarrhoea	68	3.4	2.7–4.4	2.3	4.6	**0.007**
Skin mass	63	3.2	2.5–4.1	2.8	3.7	0.252
Undesirable behaviour	52	2.6	2.0–3.4	2.7	2.6	0.994
Conjunctivitis	45	2.3	1.7–3.0	2.3	2.2	0.875
Gastroenteritis	40	2.0	1.5–2.8	1.5	2.5	0.112
Pododermatitis	40	2.0	1.5–2.8	2.1	1.8	0.623
Nails overlong	39	2.0	1.4–2.7	1.7	2.0	0.623
Pruritus	37	1.9	1.3–2.6	2.1	1.6	0.403
Tick infestation	35	1.8	1.2–2.5	1.8	1.7	0.860
Skin disorder	35	1.8	1.2–2.5	2.0	1.5	0.390
Lipoma	34	1.7	1.2–2.4	1.6	1.8	0.734
Umbilical hernia	31	1.6	1.1–2.2	2.0	1.1	0.102
Atopic dermatitis	31	1.6	1.1–2.2	1.3	1.8	0.368
Pyoderma	27	1.4	0.9–2.0	1.0	1.7	0.177
Claw injury	26	1.3	0.9–1.9	0.6	1.9	**0.009**
Haircoat disorder	26	1.3	0.9–1.9	0.9	1.6	0.160
Lameness	26	1.3	0.9–1.9	1.0	1.6	0.238
Retained deciduous tooth/teeth	25	1.3	0.8–1.9	1.0	1.5	0.317

The *P*-value reflects prevalence comparison between females and males. *P*-values that are less than 0.05 are highlighted in bold. *CI confidence interval

There were 50 distinct grouped-level precision disorder terms recorded. The most prevalent grouped-level precision disorders were dental (*n* = 378/1972, prevalence: 19.2, 95% CI: 17.5–21.0), enteropathy (270/1972, 13.7, 95% CI: 12.2–15.3), cutaneous (250/1972, 12.7, 95% CI: 11.2–14.2) and aural (197/1972, 10.0, 95% CI: 8.7–11.4). Females were more likely than males to be diagnosed with 3 of the 15 most common grouped-level precision disorders (dental, obesity and cardiac) while males had higher prevalence for enteropathy, although some of these differences were numerically small (Table 4).

**Table 4:** Prevalence of the most common *grouped-level disorders* recorded in Miniature Schnauzers (*n* = 1972) attending UK primary-care veterinary practices participating in the VetCompass™ Programme from January 1st, 2013 to December 31st, 2013

Grouped-level disorder	Count	Overall prevalence	95% CI*	Female prevalence %	Male prevalence %	*P*-value
Dental	378	19.2	17.5–21.0	21.2	17.3	**0.028**
Enteropathy	270	13.7	12.2–15.3	11.6	15.9	**0.006**
Skin	250	12.7	11.2–14.2	11.6	13.7	0.159
Aural	197	10.0	8.7–11.4	9.8	10.3	0.718
Obesity	164	8.3	7.1–9.6	10.2	6.4	**0.002**
Anal sac	128	6.5	5.4–7.7	6.0	7.0	0.367
Mass-associated	123	6.2	5.2–7.4	5.7	6.8	0.311
Ophthalmological	112	5.7	4.7–6.8	5.7	5.7	0.991
Cardiac	85	4.3	3.5–5.3	5.6	3.1	**0.005**
Neoplastic	83	4.2	3.3–5.2	3.9	4.6	0.438
Musculoskeletal	80	4.1	3.2–5.0	4.1	4.1	0.993
Parasitic	77	3.9	3.1–4.9	4.3	3.6	0.411
Claw/nail	75	3.8	3.0–4.7	3.0	4.5	0.075
Traumatic injury	70	3.5	2.8–4.5	3.0	4.2	0.147
Behavioural	65	3.3	2.6–4.2	3.0	3.7	0.382

The *P*-value reflects prevalence comparison between females and males. *P*-values that are less than 0.05 are highlighted in bold. *CI confidence interval

## Discussion

This study presents the largest analysis of demography, mortality and disorder prevalence in Miniature Schnauzers based exclusively on primary-care veterinary clinical records reported to date. The data used in this study came from 455,557 dogs from 304 clinics in the VetCompass™ database under veterinary care during 2013, including 3857 Miniature Schnauzers. This study population represents a ~5% sample of the estimated 8 million dogs in the UK [36] while the veterinary clinics represent around 6% of the estimated 5069 veterinary clinics in the UK [37]. The study can therefore be considered as highly representative of the diagnosed health of the breed in the UK. The use of electronic patient record data for epidemiological research in veterinary practice is now well-established, with a number of studies using a standardized VetCompass methodology published that report on the epidemiology of specific diseases e.g. cranial cruciate ligament disease [38], patellar luxation [39], appendicular osteoarthritis [40] and road traffic accidents [41] as well as the epidemiology of disease within specific breeds such as Rottweilers [42], Cavalier King Charles Spaniels [43], Border Terriers [44] and German Shepherd Dogs [45]. The use of “big data” from primary-care practice provides an opportunity to reveal a much more representative overview of breed health than alternative case series or surveys from teaching hospitals or referral populations [28].

This study aimed to broadly report on the health status of one breed using clinical data recorded on animals under primary veterinary care so that these data could be reasonably generalisable to the overall population of that breed in the UK and would be comparable to results for other VetCompass breed studies based on a similar methodology. Parameters for inclusion in the study population were constructed with this goal in mind. Application of a one-year period for being under veterinary care aimed to assign standardized temporal boundaries to the health window under review [46]. Permission of an open boundary-free time window during which all recorded disorders were extracted would introduce bias whereby longer lived breeds and individuals would have longer windows to contribute disorder data and therefore may appear less healthy. The inclusion and exclusion criteria for ‘dogs under veterinary care’ were designed to capture not just those animals that had visited the veterinary clinics during the year of interest but also those individuals that would also have visited if they had needed veterinary care but which were deemed as not needing such care by their owners. The results of the study should therefore be more referable to the wider population of owned dogs. Studies that include only those animals that visit the veterinary clinic are likely to be subject to selection bias towards the sicker proportion of the overall population [47]. A recent study of disease predispositions across all dog breeds has highlighted that a very large body of work has been published on the health status of the domestic dog [14]. However, despite acknowledging the value of this large volume of publications, the book also highlighted that interpretation and comparison across studies was limited by the wide variation in design elements such as in study design (e.g. cross-sectional, case-control or cohort), date, geographic location, data source (e.g. questionnaire, veterinary clinical records or insurance data), disease case definitions and reporting format. The authors of the current study aimed to overcome some of these limitations by including the current study within a series of breed-based studies based on a common data resource, taxonomy, study design, analysis and reporting structure [29]. Studies designed based on a strong and standardized core methodology should facilitate higher comparability and safer inference so that the results offer greater support for efforts by owners, breeders, veterinarians and scientists to improve breed health and welfare [42, 44, 45, 48–50].

The current study reported a relatively stable popularity for the Miniature Schnauzer breed in the general UK population over the past decade ranging from 0.7 to 1.0% off all births annually. Showing similar consistency, the Miniature Schnauzer comprised 2.0 to 2.5% of the annual registered pedigree subset of the overall population during the same period [5]. The continuing popularity of the breed may partly be because of its perception for good temperament, described as “friendly, smart, obedient” by the American Kennel Club [7]. Additionally, its small stature means it can be accommodated as a household pet more easily than larger breeds, and it is perceived to be relatively healthy [3].

Overall, 57% of the Miniature Schnauzers with available data in the current study were neutered, with females more likely to be neutered than males (62.1% versus 52.1% respectively). These values are very similar to the results for the general dog population from another UK primary-care veterinary dataset which reported that 57.1% of dogs overall were neutered, including 59.2% of females and 55.0% of males [51]. By contrast, data from the US in 2007 reported much lower uptake of neutering than in the UK for dogs overall and similar levels of neutering in females and males (33% of females vs 32% of males neutered) [52]. Decision-making on neutering by owners is complex and includes issues around health and welfare, unwanted behaviour, function of the pet, veterinary advice and financial aspects [53]. The results of the current study suggest that owners in the UK are more likely in general to have their dogs neutered than in the USA, and that owners of Miniature Schnauzers make neutering decisions that are in line with the national average for dogs overall.

The most common causes of mortality of Miniature Schnauzers in this study were neoplasia, collapse, mass-associated disorder and brain disorder. Neoplasia (14.7%) and mass-associated disorders (10.7%) may largely both reflect underlying neoplastic processes and together accounted for 25.4% of the mortality. Collapse accounted for 13.3% of deaths but underlying causes for collapse are varied and can involve neurological, orthopaedic, cardiac and other medical conditions [54]. Brain related causes accounted for 10.7% of deaths and this term again covers a range of underlying possible aetiologies [55]. A study of mortality across all purebred dogs using a similar methodology to the current study identified proportional deaths from neoplasia, collapse and neurological disease at 16.5, 3.7 and 11.2% respectively [35]. These result suggest that the Miniature Schnauzer shares a similar probability of death from neoplasia or neurological disease to the general dog population but the results do raise some questions about why the proportional death rate from collapse in the Miniature Schnauzer appears higher than for all dogs. It is worth noting, however, that the current study included only 75 deaths with a recorded cause. Furthermore, the small sample of deaths available required that the causes of death were reported at a grouped level which may have obscured some precise (fine level) causes of interest. Consequently, a much larger study with an a priori focus on mortality would be needed to explore these mortality issues more fully.

This study reported a median longevity of 11.6 years in the Miniature Schnauzer, with no difference identified between males and females. It is worth noting that the estimation of true longevity would require a cohort study design where all animals in the cohort were followed from birth until all the animals had died. Given that such a study design would be very difficult to implement, the longevity estimate reported in the current cross-sectional study is based on an assumption of a static proportional birth rate of Miniature Schnauzers over a time period equivalent to the lifetime of the breed [14]. While this assumption is violated for many breeds that have increased or decreased substantially in popularity in recent years [42, 44, 45, 48, 50], the results shown in Fig. 1 suggest that the assumption is relatively safe for the Miniature Schnauzer. This is similar to the median longevity of 12.0 years across all breeds that has been previously reported using a similar method [35]. There is substantial prior evidence in dogs that smaller breeds show increased longevity compared to larger breeds [56–58]. The mean adult bodyweight of Miniature Schnauzers in the current study was 9.9 kg. As a relatively small breed, Miniature Schnauzers may have an intrinsic longevity advantage and perhaps could have been expected to have shown greater longevity than for dogs overall assuming an average level of ‘healthiness’ in the Miniature Schnauzer compared to all dogs. However, there are many other factors that can affect longevity statistics including genetic, epigenetic and environmental effects as well as changing demography over time, and so using longevity directly as a measure of breed health is problematic and may be too simplistic [44].

Gaining a deeper understanding of disease prevalence and predisposition within breeds, and understanding the inherited component of particular predispositions, is critically important to improving overall breed health as well as raising surveillance awareness in veterinarians and owners of individual dogs [59]. For the purposes of the current paper, we have accepted increased probability of disease compared with dogs overall or with common breeds of a similar bodysize as strong evidence of disease predisposition [14]. Periodontitis describes a condition with inflammation of the ligaments and alveolar bone supporting the teeth [60]. As well as causing localised pain and tooth loss, periodontal disease is increasingly associated with systemic disease, emphasising the clinical and welfare importance of recognising and treating this condition [61]. Periodontal disease was the most prevalent disorder reported in the current study, with a prevalence of 17.4% in Miniature Schnauzers. This value is similar to the results from two studies of similar-sized breeds using the same methodology as the current study: the Cavalier King Charles Spaniel (15.2%) and the Border Terrier (17.6%) [43, 44]. Conversely, the Pug has been reported with a lower prevalence of periodontal disease (6.1%) but this value may be confounded by age: as a breed that is rapidly increasing in popularity, the average age of Pugs in that study was very young which may partially explain the low prevalence of this age-related disorder [50]. Overall, this suggests that the Miniature Schnauzer does not have a breed predisposition to periodontal disease but the high prevalence still marks out periodontal disease as a very important disease to the breed. However, despite even these relatively high prevalence values for periodontal disease, it is possible that only the more severely affected subset of dogs were identified and diagnosed during general veterinary examinations and that the true prevalence of periodontal disease may be higher still. In a research population of beagles where full-mouth examination was carried out under general anaesthesia and where periodontal disease was defined as clinical attachment loss ≥1 mm, the prevalence of periodontal disease was reported at 20% in one-year old dogs and 84% in dogs over 3 years [62]. A cross-sectional study of dogs in a commercial breeding facility in the USA reported an 86.3% prevalence of periodontal disease using examination of the dentition and gingiva without sedation or anaesthesia [63]. Nonetheless, the results from the current study highlight dental disease as a disorder priority and suggest that attention to increased dental prophylaxis for the Miniature Schnauzer by owners and veterinarians is warranted.

The second most prevalent disorder recorded at a fine level of diagnosis was obesity/overweight with a prevalence of 8.3%. This value is slightly higher than the 6.1% prevalence reported across all dogs using a similar methodology [32]. However, studies of similar sized breeds to the Miniature Schnauzer that used the same reporting methodology reported obesity/overweight prevalence values of 7.0% in the Border Terrier and 13.2% in the Pug [44, 50] suggesting that the Miniature Schnauzer does not have a breed predisposition to obesity/overweight. Obesity in dogs has been co-morbidly associated with several conditions including heart disease, insulin resistance, osteoarthritis and increased concentrations of inflammatory markers [40, 64, 65]. The high prevalence of obesity identified here for the Miniature Schnauzer, regardless of a breed predisposition, warrants enhanced owner education and veterinary monitoring to ensure healthy bodyweight targets are acknowledged and agreed. It is also important to recognise that retrospective analyses of veterinary clinical records where explicit purposive recording of obesity is not standard may underestimate the true prevalence of obesity although such studies are still very useful for reliable comparative analyses between breed [44, 66–68].

The third most prevalent disorder of Miniature Schnauzers in the current study was anal sac impaction (5.8% affected). A study across all breeds using a similar methodology reported a 7.1% prevalence for anal sac impaction across dogs overall [32] while the prevalence in the Border Terrier, Cavalier King Charles Spaniel and Pug were reported as 4.8, 3.6 and 6.5% respectively [43, 44, 50]. Anal sac impaction occurs when the anal sacs fail to empty, and can lead to uncomfortable or painful swelling of the sacs [69]. From a research perspective, anal sac disease in dogs is a classic ‘neglected disease’ that has high morbidity and welfare impact but that receives scant research interest or funding [32, 70]. Although the current study did not identify a breed predisposition to anal sac impaction in the Miniature Schnauzer, the high prevalence shown here still marks out this disorder as a potential priority for owners and veterinarians to monitor and manage to maintain good welfare in the breed.

Otitis externa was identified as affecting 5.0% of the Miniature Schnauzers in the current study. This value is less than the 10.2% prevalence reported in an earlier study of all dogs under veterinary care in the UK [32]. This compares with 6.7% in the Border Terrier, 9.2% in the Cavalier King Charles and 7.5% in the Pug [43, 44, 50]. Otitis externa is commonly encountered in primary-care clinical practice. A Canadian epidemiological study of 320 dogs undergoing routine examinations over a six-year period reported a diagnosis frequency of 15.9% for otitis externa. Schnauzers were reported to have an above-average frequency of otitis externa at 26.7%, but it was not described whether this refers to giant, standard, miniature or all Schnauzers, and the statistical significance of this increase was not reported [71]. Regular assessment of aural health should be recommended to owners of Miniature Schnauzers to identify and manage this condition early in its clinical course in order to reduce its overall negative welfare impact [72].

Information on sex predisposition to disease can assist prospective owners to select the sex that best fits their circumstances and desires, and to better manage the health of their current dog [45]. The impacts from appropriate sex selection can be highest where the sex effect is strong and the condition concerned is one that is very relevant to the owner or has major welfare implications for affected animals [45]. Some sex predispositions were noted for Miniature Schnauzers in the current study. Females were more likely to have periodontal disease, be obese/overweight and/or to have a heart murmur, while males were more likely to show diarrhoea and claw injuries.

Studies analysing veterinary primary-care clinical records have several limitations that have been reported previously [26, 42]. Clinical records are generally not recorded with research as the primary aim and therefore the data-recording processes are relatively non-standardised. The current study reported disorder terms at two levels of precision (fine level and grouped level) to take into account the varying depths of clinical precision within these data. The accuracy and detail of clinical record keeping may vary between individual veterinarians and the depth of diagnostic investigation may vary according to owner preference and financial constraints as well by veterinarian experience, expertise and access to diagnostic equipment [42]. The current study included dogs attending over 300 practices in order to reduce the effects of information bias that might accompany a study from a much smaller spread of practices. The first clinical sign listed was accepted as the disorder term for conditions that were described using multiple presenting sign terms. For combination terms where the order of the terms is not random (e.g. veterinarians may preferentially record ‘vomiting and diarrhoea’ rather than ‘diarrhoea and vomiting’), this may bias these presentations towards the preferential first term at a fine level of reporting. However, the results in the current study were also reported at a grouped level of precision to accommodate this uncertainty whereby ‘vomiting and diarrhoea’ and ‘diarrhoea and vomiting’ would both be classified as ‘enteropathy’. Diagnoses for diseases that are simpler and cheaper to identify (e.g. anal sac impaction) are more likely to be recorded than diagnoses for complex diseases (e.g. hyperadrenocorticism). Results from primary care studies such as the current study can be treated as reliable evidence sources on the overall spread of disorders that are diagnosed but it is unclear how these results may differ to the true (but unknown) levels of underlying disorders in these dogs. At a consultation level, only 20.7% of health problems in companion animals attending UK primary practices had an associated definitive diagnosis with the remaining disorders recorded descriptively using clinical sign terminology; however, the proportion of disorders recorded with a formal diagnostic term may be higher in methodologies such as used in the current study where clinical records are followed over time rather than just at the consultation level [73]. Substantial disease misclassification in clinical diagnostic reporting is suggested by a study of referral caseloads of cats and dogs in Switzerland which reported disagreement between ante-mortem and post-mortem diagnosis in 17.9% of cats and 16.0% of dogs [74]. However, the lower complexity of the usual primary-care clinical caseload suggests that the levels of diagnostic misclassification may be lower in primary-care compared with referral practice. It is increasingly recognised that veterinary primary healthcare is practiced within a very complex and nuanced setting and requires its own scholarship that is quite distinct to the second opinion setting [75]. Veterinary primary care practitioners can become very adept at applying clinical reasoning processes that can achieve excellent clinical outcomes even in the face of severe clinical uncertainly; this means that reaching a final formal diagnosis is not always, or even often, needed to gain a good clinical outcome [76, 77] The current study was geographically confined to the UK so caution should be applied when generalizing the results to other countries. Countries may differ widely in the genetics of individual breeds and the norms of veterinary clinical practice and, consequently, results from one country cannot be assumed as safe to apply to other countries without validation [78]. The current study was underpowered to report on uncommon conditions that were therefore reported with wide confidence intervals and these results should be treated with caution.

## Conclusion

Understanding breed health is complex but a basic unchanging requirement is the need for reliable health data. This study reported demographic, longevity and disease data for the general population of Miniature Schnauzers in the UK and identified periodontal disease, obesity/overweight and anal sac impaction as common diagnoses. These results can aid veterinary surgeons in diagnostic and preventive management decision-making and can guide breed organisations on evidence-based breeding reforms [79]. The study also highlights the power of primary-care veterinary clinical records for research to help understand breed health in dogs and to support evidence based approaches towards improved health and welfare in dogs.

## Abbreviations

CI: Confidence interval
EPR: Electronic patient record
IQR: Interquartile range
KC: The Kennel Club
OR: Odds ratio
PMS: Practice management systems
SD: Standard deviation

## References

[1] American Miniature Schnauzer Club: History of the Miniature Schnauzer [http://www.amsc.us/history-of-the-miniature-schnauzer/]. Accessed 30 Jan 2019.

[2] The Miniature Schnauzer Club: The Miniature Schnauzer - Early History [http://www.theminiatureschnauzerclub.co.uk/the-breed/early-history/]. Accessed 30 Jan 2019.

[3] The Kennel Club: Breed Standards Information: Dog Breeds & Groups [https://www.thekennelclub.org.uk/activities/dog-showing/breed-standards/]. Accessed 30 Jan 2019.

[4] American Kennel Club: Dog Breeds: This is the official list of all American Kennel Club dog breeds. [http://www.akc.org/breeds/index.cfm]. Accessed 30 Jan 2019.

[5] The Kennel Club: Breed registration statistics [http://www.thekennelclub.org.uk/registration/breed-registration-statistics/]. Accessed 30 Jan 2019.

[6] Anonymous (2016). Celebrations – and controversy – at the 125th Crufts dog show. Veterinary Record.

[7] American Kennel Club: Miniature Schnauzer [https://www.akc.org/dog-breeds/miniature-schnauzer/]. Accessed 30 Jan 2019.

[8] Bateson P (2010). Independent inquiry into dog breeding.

[9] Hodgman SFJ (1963). Abnormalities and defects in pedigree dogs–I. An investigation into the existence of abnormalities in pedigree dogs in the British Isles. J Small Anim Pract.

[10] Rooney NJ (2009). The welfare of pedigree dogs: cause for concern. J Vet Behav: Clin Appl Res.

[11] The Kennel Club: Breed Watch [https://www.thekennelclub.org.uk/services/public/breed/watch/Default.aspx]. Accessed 30 Jan 2019.

[12] The Kennel Club: Breed Watch Booklet 2018. London: The Kennel Club,; 2018. Available from: http://www.thekennelclub.org.uk/media/341575/breed_watch_booklet.pdf. Accessed 30 Jan 2019.

[13] Dohoo I, Martin W, Stryhn H (2009). Veterinary epidemiologic research.

[14] Gough A, Thomas A, O'Neill D (2018). Breed predispositions to disease in dogs and cats.

[15] Krieger N (1994). Epidemiology and the web of causation: has anyone seen the spider?. Soc Sci Med.

[16] Spruijt CG, Vermeulen M (2014). DNA methylation: old dog, new tricks?. Nat Struct Mol Biol.

[17] Gavazza A, Presciuttini S, Keuper H, Lubas G (2012). Estimated prevalence of canine type 2 Von Willebrand disease in the Deutsch-Drahthaar (German wirehaired pointer) in Europe. Res Vet Sci.

[18] Teng KT, McGreevy PD, Toribio J-ALML, Dhand NK (2016). Trends in popularity of some morphological traits of purebred dogs in Australia. Canine Genetics Epidemiol.

[19] Birkegård AC, Reimann MJ, Martinussen T, Häggström J, Pedersen HD, Olsen LH (2016). Breeding restrictions decrease the prevalence of myxomatous mitral valve disease in cavalier king Charles spaniels over an 8- to 10-year period. J Vet Intern Med.

[20] Mori N, Lee P, Muranaka S, Sagara F, Takemitsu H, Nishiyama Y (2010). Predisposition for primary hyperlipidemia in miniature schnauzers and Shetland sheepdogs as compared to other canine breeds. Res Vet Sci.

[21] Milne KL, Hayes HM (1981). Epidemiologic features of canine hypothyroidism. Cornell Vet.

[22] Catchpole B, Kennedy LJ, Davison LJ, Ollier WER (2008). Canine diabetes mellitus: from phenotype to genotype. J Small Anim Pract.

[23] Toulza O, Center SA, Brooks MB, Erb HN, Warner KL, Deal W (2006). Evaluation of plasma protein C activity for detection of hepatobiliary disease and portosystemic shunting in dogs. J Am Vet Med Assoc.

[24] Bartlett PC, Van Buren JW, Neterer M, Zhou C (2010). Disease surveillance and referral bias in the veterinary medical database. Prev Vet Med.

[25] Egenvall A, Nødtvedt A, Penell J, Gunnarsson L, Bonnett BN (2009). Insurance data for research in companion animals: benefits and limitations. Acta Vet Scand.

[26] O'Neill D, Church D, McGreevy P, Thomson P, Brodbelt D (2014). Approaches to canine health surveillance. Canine Genetics Epidemiol.

[27] Collins LM, Asher L, Summers JF, Diesel G, McGreevy PD (2010). Welfare epidemiology as a tool to assess the welfare impact of inherited defects on the pedigree dog population. Anim Welf.

[28] McGreevy PD, Nicholas FW (1999). Some practical solutions to welfare problems in dog breeding. Anim Welf.

[29] VetCompass: VetCompass: Health surveillance for UK companion animals [http://www.rvc.ac.uk/VetCOMPASS/]. Accessed 30 Jan 2019.

[30] Pearce N (2012). Classification of epidemiological study designs. Int J Epidemiol.

[31] Epi Info 7 CDC: Centers for Disease Control and Prevention (US): Introducing Epi Info 7 [http://wwwn.cdc.gov/epiinfo/7]. Accessed 30 Jan 2019.

[32] O'Neill DG, Church DB, McGreevy PD, Thomson PC, Brodbelt DC (2014). Prevalence of disorders recorded in dogs attending primary-care veterinary practices in England. PLoS One.

[33] Kirkwood BR, Sterne JAC (2003). Essential Medical Statistics.

[34] Agresti A, Coull BA (1998). Approximate is better than “exact” for interval estimation of binomial proportions. Am Stat.

[35] O'Neill DG, Church DB, McGreevy PD, Thomson PC, Brodbelt DC (2013). Longevity and mortality of owned dogs in England. Vet J.

[36] Asher L, Buckland E, Phylactopoulos CL, Whiting M, Abeyesinghe S, Wathes C (2011). Estimation of the number and demographics of companion dogs in the UK. BMC Vet Res.

[37] RCVS (2012). RCVS facts 2012: part 1.

[38] Taylor-Brown FE, Meeson RL, Brodbelt DC, Church DB, McGreevy PD, Thomson PC (2015). Epidemiology of cranial cruciate ligament disease diagnosis in dogs attending primary-care veterinary practices in England. Vet Surg.

[39] O'Neill DG, Meeson RL, Sheridan A, Church DB, Brodbelt DC (2016). The epidemiology of patellar luxation in dogs attending primary-care veterinary practices in England. Canine Genetics Epidemiol.

[40] Anderson KL, O'Neill DG, Brodbelt DC, Church DB, Meeson RL, Sargan D (2018). Prevalence, duration and risk factors for appendicular osteoarthritis in a UK dog population under primary veterinary care. Sci Rep.

[41] Harris GL, Brodbelt D, Church D, Humm K, McGreevy PD, Thomson PC (2018). Epidemiology, clinical management, and outcomes of dogs involved in road traffic accidents in the United Kingdom (2009–2014). J Vet Emerg Crit Care.

[42] O'Neill DG, Seah WY, Church DB, Brodbelt DC (2017). Rottweilers under primary veterinary care in the UK: demography, mortality and disorders. Canine Genetics Epidemiol.

[43] Summers J, O'Neill D, Church D, Thomson P, McGreevy P, Brodbelt D (2015). Prevalence of disorders recorded in cavalier king Charles spaniels attending primary-care veterinary practices in England. Canine Genetics Epidemiol.

[44] O'Neill DG, Darwent EC, Church DB, Brodbelt DC (2017). Border terriers under primary veterinary care in England: demography and disorders. Canine Genetics Epidemiol.

[45] O'Neill DG, Coulson NR, Church DB, Brodbelt DC (2017). Demography and disorders of German shepherd dogs under primary veterinary care in the UK. Canine Genetics Epidemiol.

[46] Faybush EM, Blanchard JF, Rawsthorne P, Bernstein CN (2002). Generational differences in the age at diagnosis with IBD: genetic anticipation, bias, or temporal effects. Am J Gastroenterol.

[47] Westreich D (2012). Berkson’s bias, selection bias, and missing data. Epidemiology (Cambridge, Mass).

[48] O'Neill DG, Baral L, Church DB, Brodbelt DC, Packer RMA (2018). Demography and disorders of the French bulldog population under primary veterinary care in the UK in 2013. Canine Genetics Epidemiol.

[49] McGreevy PD, Wilson BJ, Mansfield CS, Brodbelt DC, Church DB, Dhand N (2018). Labrador retrievers under primary veterinary care in the UK: demography, mortality and disorders. Canine Genetics Epidemiol.

[50] O'Neill DG, Darwent EC, Church DB, Brodbelt DC (2016). Demography and health of pugs under primary veterinary care in England. Canine Genetics Epidemiol.

[51] Sánchez-Vizcaíno F, Noble P-JM, Jones PH, Menacere T, Buchan I, Reynolds S (2017). Demographics of dogs, cats, and rabbits attending veterinary practices in Great Britain as recorded in their electronic health records. BMC Vet Res.

[52] Trevejo R, Yang M, Lund EM (2011). Epidemiology of surgical castration of dogs and cats in the United States. J Am Vet Med Assoc.

[53] Downes MJ, Devitt C, Downes MT, More SJ (2015). Neutering of cats and dogs in Ireland; pet owner self-reported perceptions of enabling and disabling factors in the decision to neuter. PeerJ.

[54] Taylor SM, Shmon CL, Shelton GD, Patterson EE, Minor K, Mickelson JR (2008). Exercise-induced collapse of Labrador retrievers: survey results and preliminary investigation of heritability. J Am Anim Hosp Assoc.

[55] Gough A, Murphy K (2015). Differential Diagnosis in Small Animal Medicine.

[56] Galis F, Van Der Sluijs I, Van Dooren TJM, Metz JAJ, Nussbaumer M (2007). Do large dogs die young?. J Exp Zool B Mol Dev Evol.

[57] Greer KA, Canterberry SC, Murphy KE (2007). Statistical analysis regarding the effects of height and weight on life span of the domestic dog. Res Vet Sci.

[58] Patronek GJ, Waters DJ, Glickman LT (1997). Comparative longevity of pet dogs and humans: implications for gerontology research. J Gerontol: Biological Sci.

[59] Farrell L, Schoenebeck J, Wiener P, Clements D, Summers K (2015). The challenges of pedigree dog health: approaches to combating inherited disease. Canine Genetics Epidemiol.

[60] Gulati M, Anand V, Jain N, Anand B, Bahuguna R, Govila V (2013). Essentials of periodontal medicine in preventive medicine. Int J Prev Med.

[61] Rawlinson JE, Goldstein RE, Reiter AM, Attwater DZ, Harvey CE (2011). Association of periodontal disease with systemic health indices in dogs and the systemic response to treatment of periodontal disease. J Am Vet Med Assoc.

[62] Kortegaard H, Eriksen T, Baelum V (2014). Screening for periodontal disease in research dogs - a methodology study. Acta Vet Scand.

[63] Stella JL, Bauer AE, Croney CC (2018). A cross-sectional study to estimate prevalence of periodontal disease in a population of dogs (Canis familiaris) in commercial breeding facilities in Indiana and Illinois. PLoS One.

[64] Thengchaisri N, Theerapun W, Kaewmokul S, Sastravaha A (2014). Abdominal obesity is associated with heart disease in dogs. BMC Vet Res.

[65] Tropf M, Nelson OL, Lee PM, Weng HY (2017). Cardiac and metabolic variables in obese dogs. J Vet Intern Med.

[66] Lund EM, Armstrong PJ, Kirk CA, Klausner JS (2006). Prevalence and risk factors for obesity in adult dogs from private US veterinary practices. Int J Applied Res Vet Med.

[67] McGreevy PD, Thomson PC, Pride C, Fawcett A, Grassi T, Jones B (2005). Prevalence of obesity in dogs examined by Australian veterinary practices and the risk factors involved. Vet Rec.

[68] Robertson ID (2003). The association of exercise, diet and other factors with owner-perceived obesity in privately owned dogs from metropolitan Perth. WA Prevent Vet Med.

[69] van Duijkeren E (1995). Disease conditions of canine anal sacs. J Small Anim Pract.

[70] Moran M, Guzman J, Ropars A-L, McDonald A, Jameson N, Omune B (2009). Neglected disease Research and Development: how much are we really spending?. PLoS Med.

[71] Perry LR, MacLennan B, Korven R, Rawlings TA (2017). Epidemiological study of dogs with otitis externa in cape Breton, Nova Scotia. Can Vet J.

[72] Jacobson L (2002). Diagnosis and medical treatment of otitis externa in the dog and cat: review article. J S Afr Vet Assoc.

[73] Robinson NJ, Dean RS, Cobb M, Brennan ML (2016). Factors influencing common diagnoses made during first-opinion small-animal consultations in the United Kingdom. Prev Vet Med.

[74] Schertenleib TI, Pospischil A, Hässig M, Kircher PR, Hilbe M (2017). Comparison of clinical and pathological diagnoses in cats and dogs. J Comp Pathol.

[75] May S (2015). Towards a scholarship of primary health care. Vet Rec.

[76] Wilson T, Holt T, Greenhalgh T (2001). Complexity and clinical care. BMJ.

[77] Vandeweerd J-M, Vandeweerd S, Gustin C, Keesemaecker G, Cambier C, Clegg P (2012). Understanding veterinary practitioners' decision-making process: implications for veterinary medical education. J Vet Med Educ.

[78] Takeuchi Y, Mori Y (2006). A comparison of the behavioral profiles of purebred dogs in Japan to profiles of those in the United States and the United Kingdom. J Vet Med Sci.

[79] The Kennel Club: The Kennel Club’s Breed Health and Conservation Plans project [https://www.thekennelclub.org.uk/health/breed-health-and-conservation-plans/]. Accessed 30 Jan 2019.

